# Immune-Related Genes Are Prognostic Markers for Prostate Cancer Recurrence

**DOI:** 10.3389/fgene.2021.639642

**Published:** 2021-08-19

**Authors:** Min Fu, Qiang Wang, Hanbo Wang, Yun Dai, Jin Wang, Weiting Kang, Zilian Cui, Xunbo Jin

**Affiliations:** ^1^Department of Urology, Shandong Provincial Hospital Affiliated to Shandong First Medical University, Jinan, China; ^2^Department of Urology, Shandong Provincial Hospital, Cheeloo College of Medicine, Shandong University, Jinan, China; ^3^Department of Human Resources, Shandong Provincial Hospital Affiliated to Shandong First Medical University, Jinan, China; ^4^Department of Ultrasound, Shandong Provincial Hospital Affiliated to Shandong First Medical University, Jinan, China; ^5^Department of Ultrasound, Shandong Provincial Hospital, Cheeloo College of Medicine, Shandong University, Jinan, China; ^6^Department of Urology, The First Affiliated Hospital of Shandong First Medical University, Jinan, China; ^7^Department of Urology, Shandong Provincial Qianfoshan Hospital, Cheeloo College of Medicine, Shandong University, Jinan, China

**Keywords:** prostate cancer, weighted gene co-expression network analysis, immune-related genes, LASSO Cox regression, immune infiltration, nomogram

## Abstract

**Background:**

Prostate cancer (PCa) is an immune-responsive disease. The current study sought to explore a robust immune-related prognostic gene signature for PCa.

**Methods:**

Data were retrieved from the tumor Genome Atlas (TCGA) database and GSE46602 database for performing the least absolute shrinkage and selection operator (LASSO) cox regression model analysis. Immune related genes (IRGs) data were retrieved from ImmPort database.

**Results:**

The weighted gene co-expression network analysis (WGCNA) showed that nine functional modules are correlated with the biochemical recurrence of PCa, including 259 IRGs. Univariate regression analysis and survival analysis identified 35 IRGs correlated with the prognosis of PCa. LASSO Cox regression model analysis was used to construct a risk prognosis model comprising 18 IRGs. Multivariate regression analysis showed that risk score was an independent predictor of the prognosis of PCa. A nomogram comprising a combination of this model and other clinical features showed good prediction accuracy in predicting the prognosis of PCa. Further analysis showed that different risk groups harbored different gene mutations, differential transcriptome expression and different immune infiltration levels. Patients in the high-risk group exhibited more gene mutations compared with those in the low-risk group. Patients in the high-risk groups showed high-frequency mutations in TP53. Immune infiltration analysis showed that M2 macrophages were significantly enriched in the high-risk group implying that it affected prognosis of PCa patients. In addition, immunostimulatory genes were differentially expressed in the high-risk group compared with the low-risk group. BIRC5, as an immune-related gene in the prediction model, was up-regulated in 87.5% of prostate cancer tissues. Knockdown of BIRC5 can inhibit cell proliferation and migration.

**Conclusion:**

In summary, a risk prognosis model based on IGRs was developed. A nomogram comprising a combination of this model and other clinical features showed good accuracy in predicting the prognosis of PCa. This model provides a basis for personalized treatment of PCa and can help clinicians in making effective treatment decisions.

## Introduction

Prostate cancer is the most common genitourinary tumor in men. It is the fifth leading cause of cancer mortalities worldwide. Approximately 1.3 million new cases and 359,000 cancer-related mortalities were reported in Bray et al. (2018). Treatment modalities for prostate cancer mainly include surgical resection, radiotherapy, and hormonal therapy. The 5-year survival rate of local PCa can reach 100% owing to advances in diagnostic and therapeutic methods, however, the 5-year survival rate of metastatic PCa is approximately 30% (Litwin and Tan, 2017). Therapeutic efficacy of prostate cancer can be improved by understanding the molecular mechanisms of prostate cancer occurrence and progression.

Recently, novel therapies that modulate the immune system have been used in patients with various malignancies. These immune therapies significantly improve cancer prognosis. However, this therapy is not effective in all tumors (Goswami et al., 2016). Prostate cancer is an immune response disease. A phase III clinical trial included men with castration-resistant prostate cancer (CRPC) who progressed after docetaxel chemotherapy received radiotherapy and bone metastasis therapy with ipilimumab (CTLA-4’s antibody) or placebo. In the trial, ipilimumab prolonged the median overall survival (OS) of specific subgroups of patients lacking visceral disease (Kwon et al., 2014). Notably, combinations of ipilimumab with the prostate cancer vaccine showed more beneficial effects (Madan et al., 2012; Kwon et al., 2014). Expression of various tumor associated antigens are associated with favorable effects of immunotherapies. However, the tumor microenvironment in prostate cancer is relatively immunosuppressive and may be responsible for the failures of various agents targeting the immune system (Maia and Hansen, 2017). Phase I clinical trials showed that nivolumab does not exhibit any clinical effects in metastatic CRPC patients (Topalian et al., 2012). Therefore, an analysis of the relationships between immune genes and prostate cancer prognosis and establishment of diagnostic and prognostic immune characteristics are important to identify an effective marker for early detection and prediction of survival outcomes.

In the current study, the weighted gene co-expression network analysis method was used to identify highly correlated immune genes with prostate cancer recurrence. Genes correlated with prognosis were screened from 259 immune-related genes in the TCGA dataset. Identified genes were used for Least Absolute Shrinkage and Selection Operator (LASSO) analysis to establish the best risk model. The prediction value of the model was verified using the GSE46602 dataset. Patients were divided into high-risk and low-risk groups. Differences in gene mutations, transcription levels, and immune infiltrations in the high- and low-risk groups were analyzed. Further, a nomogram was built based on clinical features and prognostic gene signatures for prediction of survival outcomes of patients. In the current study, immune-related prognostic markers and potential mechanisms of prostate cancer were explored. The findings of this study provide a basis for subsequent personalized prostate cancer diagnosis and treatment.

## Materials and Methods

### Data Search and Preprocessing

GSE46602 expression and clinical dataset (a total of 50 cases; 36 cases were prostate cancer tissue samples whereas 14 were normal tissue samples) were obtained from the Gene Expression Omnibus (GEO)^1^ database (Barrett et al., 2011). R software was used to convert probes into gene symbols and entriz IDs. Probes that did not match any gene symbol or entriz ID were excluded from the study. If a gene symbol corresponded to multiple probes, the maximum value of the probe was used as the final expression value of the gene symbol in subsequent analysis. Probes that corresponded to multiple gene symbols were excluded from the study. A total of 1,811 immune-related genes (IRG) were retrieved the ImmPort database^2^.

### Weighted Gene Co-expression Network Analysis

The WGCNA package (Langfelder and Horvath, 2008) in R was used for construction of gene co-expression networks. Correlations between each module and prostate cancer were used to select the module that was relevant to external biological parameters. This module selected was the key module during this research. Gene expression matrix was established for genes that were 75% expressed before the median absolute deviation and whose MAD was greater than 0.01. In addition, missing values were processed. The resulting matrix was analyzed using WGCNA. The soft threshold used in this study was greater than 0.85. During the screening process, an outlier was found and removed after which, the matrix was used to construct the WGCNA network. WGCNA analysis was used to identify the modules related to the biochemical recurrence of prostate cancer, and IRGs.

### Identification and Validation of the Prognostic Gene Signature

Univariate Cox regression and survival analyses were performed using the TCGA database and the findings used to establish a prognostic model. The prognostic model was used to identify IRGs correlated with the prognosis of prostate cancer.

The prognostic gene signature was then verified. Prostate cancer patients in TCGA were randomly divided into the training cohort and the testing cohort based on a ratio of 7:3, respectively. The “glmnet” package was used to construct a multivariate model with immune-related genes using the LASSO Cox regression method in the training cohort (Sauerbrei et al., 2007; Friedman et al., 2010). LASSO regression is a variable selection method for fitting high-dimensional generalized linear models. Over-fitting can be effectively avoided by constructing a penalty function to reduce the number of variables. This leads to creation of a more refined model. The risk score for each patient was calculated using the equation risk scores = ∑ (βi × Expi), where βi represents the coefficient and Expi represents the relative expression value of the gene. In addition, the cut-off value of the training group was calculated and patients were divided into high and low risk groups based on the cut-off value. Performance of the model was evaluated using a testing set whereas external verification was performed using the GEO database.

### Differential Expression Analysis

Differentially expressed genes between high and low risk groups were determined using the limma package (Ritchie et al., 2015). Differential gene screening threshold was set at | LogFC | > 1 and *p* < 0.05.

### Gene Set Enrichment Analysis (GSEA)

Gene set enrichment analysis was performed using “clusterProfiler” package (Yu et al., 2012). Gene sets from the high- and low- risk groups were compared. Curated KEGG gene sets used in this study were retrieved from the Molecular Signature Database (c2: curated gene sets, KEGG gene sets, gene symbols)^3^.

### Evaluation of the Immune Status Between High-Risk and Low-Risk Groups Stratified by Prognostic Model

Potential relationships between the immune cell infiltration and IRGs signatures were explored using the CIBERSORTx online tool^4^ (Newman et al., 2019). This tool analyzes level of immune cell infiltration in different samples. RNA expression profiles in the TCGA dataset were used for immune-infiltration analysis. CIBERSORT was used to calculate the *p*-value of the deconvolution for each sample using the Monte Carlo sampling approach. This provided confidence in the estimation (Newman et al., 2019). Samples with *p* < 0.05 were selected for further analysis.

### Development of a Nomogram

The “survival” and the “rms” packages in R were used to construct a nomogram based on age, T stage, N stage, and risk scores. Calibration curves were then plotted to evaluate the concordance between actual and predicted survival outcomes.

### Collection of Clinical Tissue Samples

A total of 40 pairs of PCa tissue and matched normal tissues samples were obtained from the Department of Urology, Shandong Provincial Hospital, between May 2014 and June 2020. All 34 pairs of PCa tissues and matched normal tissues were used for qPCR analysis. All patients signed informed consent form, and the study was approved by the Ethics Committee of Shandong Provincial Hospital (Jinan, China).

### RNA Isolation, Reverse Transcription and Quantitative Real-Time PCR

Total RNA was extracted from prepared tissues and cells using RNAiso Plus (Takara, Japan). Total RNA was reverse-transcribed into complementary DNA (cDNA) using the PrimeScript RT kit (Takara). BIRC5 expression level was determined by qPCR reactions using the following primer sequences: forward, 5′-CAACCGGACGAATGCTTTT-3′; reverse, 5′- AAGAACTGGCCCTTCTTGGA-3′ using the SYBR Premix Ex Tap (Takara) on the LightCycler 480II (Roche, Switzerland). GAPDH mRNA was used as the internal control. All experimental procedures were carried out following the manufacturer’s protocols. Expression profiles obtained from qPCR results were analyzed using ‘pcr’ R package.

### Cell Transfection

siRNA and negative control siRNA vectors targeting BIRC5 were purchased from Sangon Biotech (Shanghai, China). The sequences were: Control siRNA: 5′-UUCUCCGAACGUGUCACGUTT-3′; BIRC siRNA: 5′- ACCGCATCTCTACATTCAA-3′. The vectors were transfected into DU145 and PC-3 cells using Lipofectamine 3000 (Invitrogen) following the manufacturer’s protocol. All transfection experiments were carried out within a period of 48 h. Efficiency of transfection was determined using qPCR analysis.

### Cell Counting Kit-8 (CCK-8) Assay

Prostate cancer (PCa) cells were seeded in a 96-well plate, and the proliferation rate was evaluated using CCK-8 assay (Dojindo, Japan). Cells were cultured for 0, 24, 48, 72, and 96 h, and 10 μL of CCK-8 was added to each well. Absorbance at 450 nm was determined with the Spectrophotometer Multiskan Go (Thermo Fisher Scientific, Finland).

### Wound Healing Assay

Prostate cancer (PCa) cells were seeded in a 6-well plate. When the cells reached confluence, a sterile micropipette tip was used to gently scrap the surface of the plate. Cells were incubated at 37°C with 5% CO_2_ and imaged at 0, 24, and 48 h. An inverted optical microscope was used to monitor wound closure. All experiments were performed in triplicates for each group.

### Statistical Analysis

Statistical analyses were performed using R software (version 3.6.1)^5^. Statistical significance was set at *p* < 0.05. Survival data were analyzed using Kaplan-Meier curve whereas univariate cox regression analysis was used to explore factors associated with patient survival. Multivariate Cox regression analysis was used to determine independent prognostic factors. Time-dependent ROC analysis was used to evaluate the accuracy of the prognostic prediction model. AUC > 0.60 was considered accurate for prediction, whereas AUC > 0.75 was considered to have excellent predictive value (Han et al., 2018; Cho et al., 2019). The “maftools” R package (Mayakonda et al., 2018) was used for visualization of gene mutations in the high- and low-risk score groups of PCa. Statistical significance was presented as ^∗^*p* < 0.05, ^∗∗^*p* < 0.01, ^∗∗∗^*p* < 0.001.

## Results

### Construction of Weighted Co-expression Network and Identification of Key Modules

**FIGURE 1:**
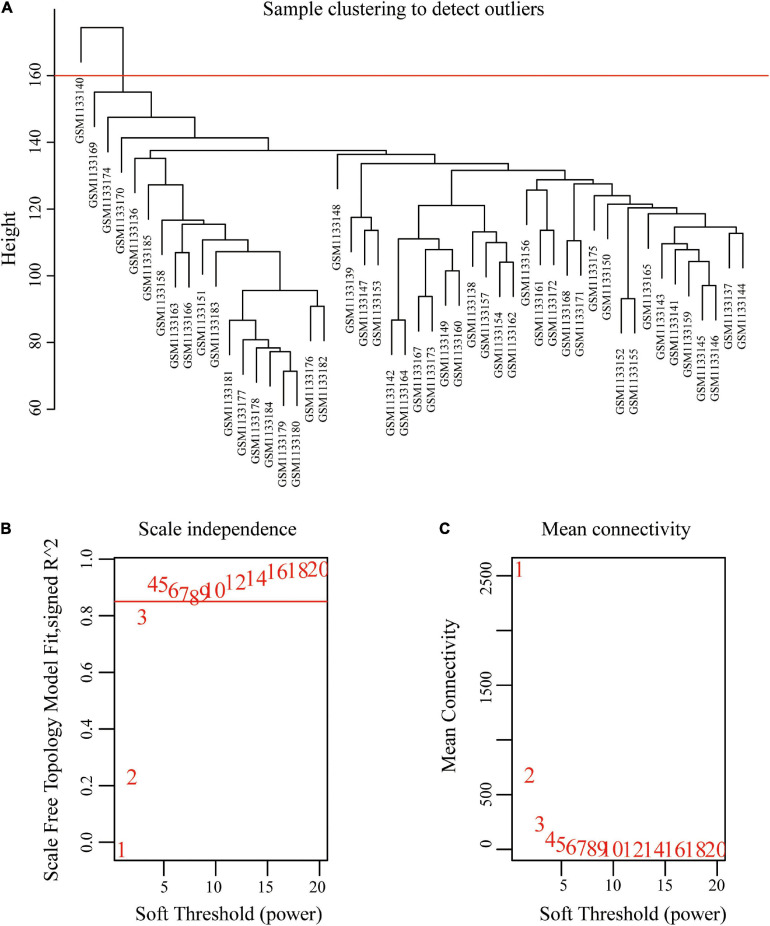
Sample clustering to detect outliers and determine soft-threshold power. **(A)** Clustering based on expression data of GSE46602, with 160 as the threshold, one outlier was removed. **(B)** Analysis of the scale-free fit index for various soft-threshold powers. **(C)** Analysis of the mean connectivity for various soft-threshold powers.

**FIGURE 2:**
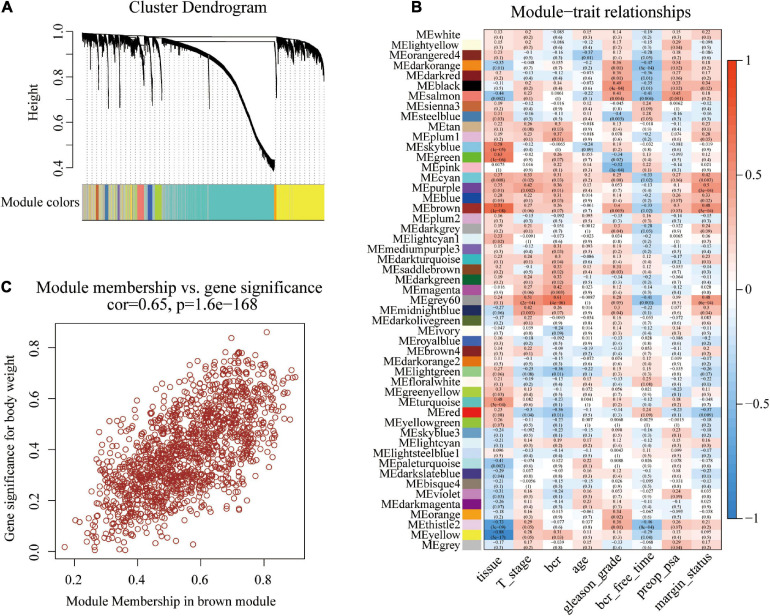
Construction of co-expression modules using the WGCNA package. **(A)** Cluster dendrogram of genes in GSE46602 dataset. Each branch in the figure represents one gene, and every color represents one co-expression module. **(B)** Heatmap of the correlation between module eigengenes and the disease status of PCa. **(C)** Scatter plot of module eigengenes in the brown module.

The original sample outlier test is presented in Figure 1A. The findings show that 160 was used as the threshold value and one outlier was excluded. The screened WGCNA soft threshold is presented in Figures 1B,C. The soft threshold value for reliable outcomes used in the WGCNA analysis was 4. A one-step method was used to construct the WGCNA network and a hierarchical clustering tree to visualize the network (Figure 2A). A total of 51 gene modules were obtained. Gray color indicates that genes are not classified into any modules, therefore, they were not included in any functional modules. At last, a total of 50 functionalmodules were obtained (Figure 2B). Yellow (*r* = −0.29, *p* = 0.04), thistle2 (*r* = −0.46, *p* = 8e-04), gray60 (*r* = −0.41, *p* = 0.003), brown (*r* = −0.33, *p* = 0.02), cyan (*r* = −0.33, *p* = 0.02), salmon (*r* = −0.41, *p* = 0.004), black (*r* = −0.35, *p* = 0.01), dark-red (*r* = −0.36, *p* = 0.01), and dark-orange (*r* = −0.47, *p* = 6e-04) modules were associated with prostate cancer recurrence. These modules comprised a total of 260 immune-related genes. Out of the 260 immune-related genes, 259 were common between GSE46602 dataset, and TCGA prostate cancer dataset. Expression levels of these genes are shown in Supplementary Tables 1, 2. In addition, the brown modules were analyzed and the findings showed that the scatter plot of the gene of importance in the brown module and the membership of the module are highly correlated (Figure 2C). This indicated that the WGCNA network was highly reliable.

### Screening Prognostic Related IRGs

The prognostic status and recurrence time for 427 prostate cancer patients were screened using the TCGA data set. A total of 35 IRGs were associated with prostate cancer prognosis (Supplementary Table 3). The 35 IRGs were then used for model construction.

### Construction and Internal Validation of Prognostic Signatures

Patients in the TCGA dataset were randomly assigned to the TCGA-training set (*n* = 301) and TCGA-testing set (*n* = 126) using a 7: 3 ratio. Clinical data of patients in the two groups is presented in Table 1. Analysis showed no statistical difference in age, stage, lymph node metastasis, and distant metastasis between the two groups (Table 1, *p* > 0.05). Therefore, another model was constructed.

**TABLE 1:** Clinical characteristics of prostate cancer patients in different datasets.

Characteristic		TCGA cohort			Validation cohort
		Training set	Testing set	*p*-Value	GSE46602
No. of samples		301	126		36
Median age in years (range)		62(41–77)	61(46–78)	0.688	63(46–71)
T	T2	104	50	0.7460	19
	T3	187	72		17
	T4	6	2		0
	NA	4	2		0
N	N0	214	90	0.537	NA
	N1	51	17		NA
	NA	36	19		NA
M	M0	281	120	0.708	NA
	M1	3	0		NA
	NA	17	6		NA
Gleason		NA	NA		7(4–9)

**FIGURE 3:**
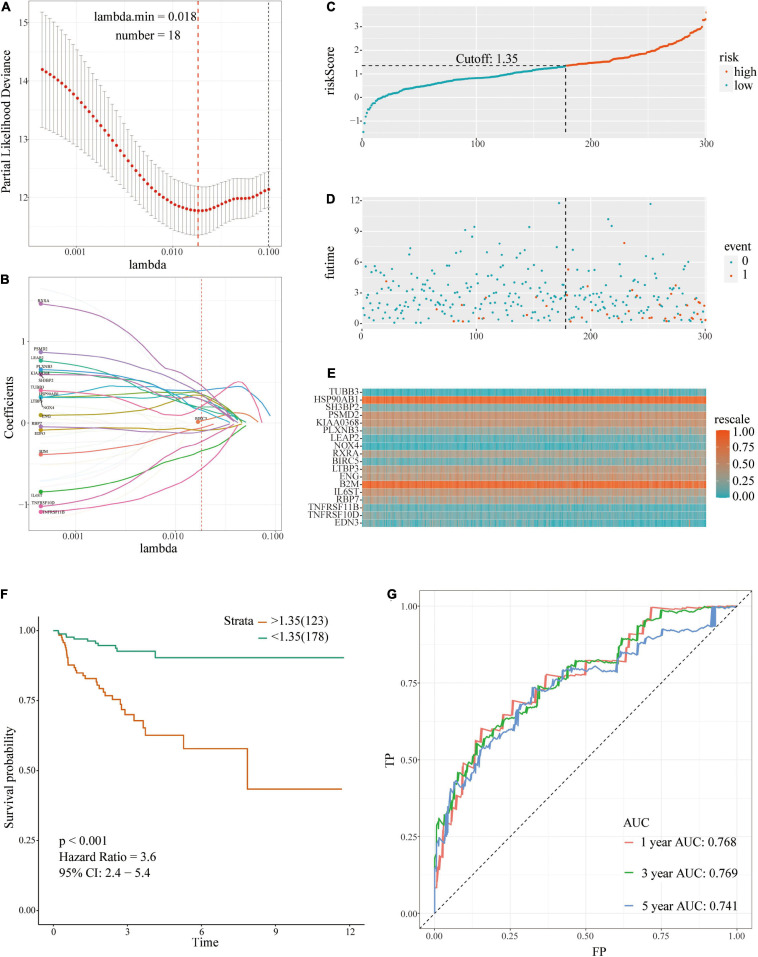
A prognostic signature based on IRGs in the TCGA-training set. **(A,B)** Identification of 18 IRGs by LASSO regression analysis; **(C)** Distribution of risk scores based on IRGs in PCa; **(D)** Recurrence status of patients in different groups; **(E)** Heatmap showing the expression profiles of IRGs; **(F)** Survival analysis for the signature-defined risk groups; **(G)** Time-dependent ROC curve of the 18-IRGs prognostic signature.

The 35 prognosis-related IRGs were used in LASSO regression analysis to identify the best prognostic immune genes and to develop a risk prognosis model. A prognostic model comprising eighteen genes (TUBB3, HSP90AB1, SH3BP2, PSMD2, KIAA0368, PLXNB3, LEAP2, NOX4, RXRA, BIRC5, LTBP3, ENG, B2M, IL6ST, RBP7, TNFRSF11B, TNFRSF10D, and EDN3) was established (Table 2 and Figures 3A,B). This model was used to evaluate outcomes in each patient. The following formula was used: risk score = (0.2081 ^∗^ expression level of TUBB3) + (0.3556 ^∗^ expression level of HSP90AB1) + (0.2891 ^∗^ expression level of SH3BP2) + (0.3530 ^∗^ expression level of PSMD2) + (0.1975 ^∗^ expression level of KIAA0368) + (0.3988 ^∗^ expression level of PLXNB3) + (0.1806 ^∗^ expression level of LEAP2) + (0.1568 ^∗^ expression level of NOX4) + (0.4577 ^∗^ expression level of RXRA) + (0.0282 ^∗^ expression level of BIRC5) + (0.2874 ^∗^ expression level of LTBP3) + (0.3368 ^∗^ expression level of ENG) + (−0.057 ^∗^ expression level of B2M) + (−0.3392 ^∗^ expression level of IL6ST) + (−0.1028 ^∗^ expression level of RBP7) + (−0.5578 ^∗^ expression level of TNFRSF11B) + (−0.2254 ^∗^ expression level of TNFRSF10D) + (−0.0886 ^∗^ expression level of EDN3). The optimal cut-off value for patients in the high- or low-risk groups was set at 1.35 after calculating the risk score of each patient in the TCGA-training set (Figure 3C).

**TABLE 2:** Model information about IRGs.

Genes	HR	95% CI	*p-*Value	Lasso coefficient
TUBB3	5.2	(2.6–11)	6.10E-06	0.208114746
HSP90AB1	4.4	(1.7–12)	0.0022	0.355584809
SH3BP2	3.8	(1.7–8.2)	9.00E-04	0.289072047
PSMD2	3.5	(1.6–7.7)	0.0016	0.352964905
KIAA0368	3.3	(1.6–6.8)	0.0015	0.197459936
PLXNB3	3	(1.9–4.9)	9.20E-06	0.398784703
LEAP2	2.9	(1.5–5.5)	0.0015	0.180642675
NOX4	2.8	(1.4–5.5)	0.0028	0.156824163
RXRA	2.3	(1.1–4.6)	0.024	0.457674926
BIRC5	1.9	(1.3–2.7)	0.00026	0.028173231
LTBP3	1.9	(1.1–3.4)	0.019	0.287378587
ENG	1.7	(1.1–2.7)	0.015	0.33681419
B2M	0.66	(0.46–0.96)	0.028	−0.057436744
IL6ST	0.6	(0.41–0.88)	0.0091	−0.339150684
RBP7	0.54	(0.37–0.78)	0.001	−0.102821028
TNFRSF11B	0.42	(0.18–1)	0.057	−0.557788482
TNFRSF10D	0.39	(0.19–0.79)	0.0086	−0.225400728
EDN3	0.3	(0.14–0.63)	0.0015	−0.088630127

The risk plot with the distribution of the patients in the groups based on the signature, prognostic status of individuals between groups, and expression levels of the included IRGs is presented in Figures 3C–E. A significant difference in the prognostic status between risk score groups was observed (red dots represent recurrence whereas blue dots represent absence of recurrence). Level of tumor recurrence in the high-risk score group was significantly higher compared with that of the low-risk score group [*p* < 0.001; HR = 3.6 (2.4, 5.4); Figure 3F]. The risk score stratified the TCGA-training set accurately and divided patients into low- and high-risk score groups based on tumor recurrence. ROC curve analysis (Figure 3G) showed good discrimination with AUCs of 0.768, 0.769, and 0.741 after 1-, 3-, and 5-year follow-up, respectively.

**FIGURE 4:**
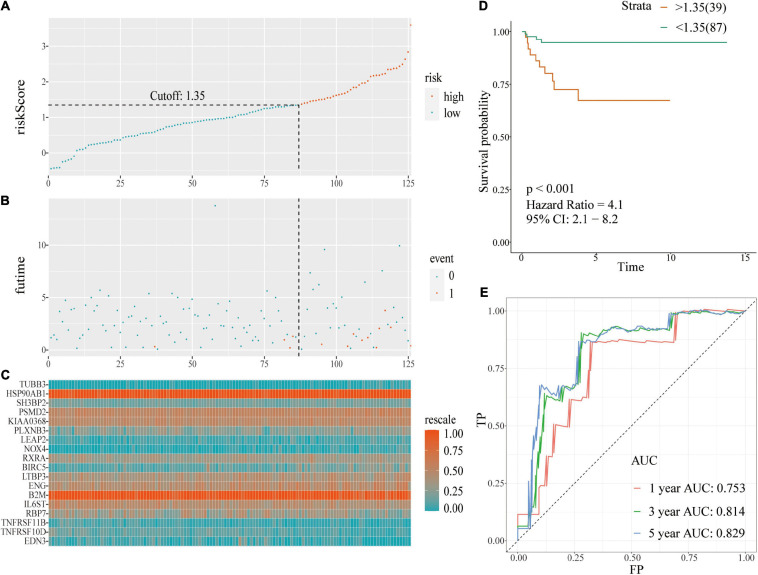
A prognostic signature based on IRGs in the TCGA-testing set. **(A)** Risk score distribution based on IRGs in PCa; **(B)** Recurrence status of patients in different groups; **(C)** Heatmap showing the expression profiles of IRGs; **(D)** Survival analysis for the signature-defined risk groups; **(E)** Time-dependent ROC curve of the 18-IRGs prognostic signature.

Risk score evaluation of PCa patients in the TCGA-testing set was performed using coefficient and relative expression value of the gene. Patients in the TCGA-testing set were classified into high- and low-risk score groups based on cut-off value. The risk plot with patient distribution based on signature, prognostic status of individuals between groups, and the expression level of included IRGs is presented in Figures 4A–C. The findings showed that tumor recurrence was significant higher in the high-risk score group compared with the low risk group [*p* < 0.001; HR = 4.1 (2.1, 8.2)] (Figure 4D). ROC curve analysis (Figure 4E) showed good discrimination with AUCs of 0.753, 0.814, and 0.829 after 1-, 3-, and 5-year follow-up, respectively.

### External Validation Using GSE46602 Datasets

**FIGURE 5:**
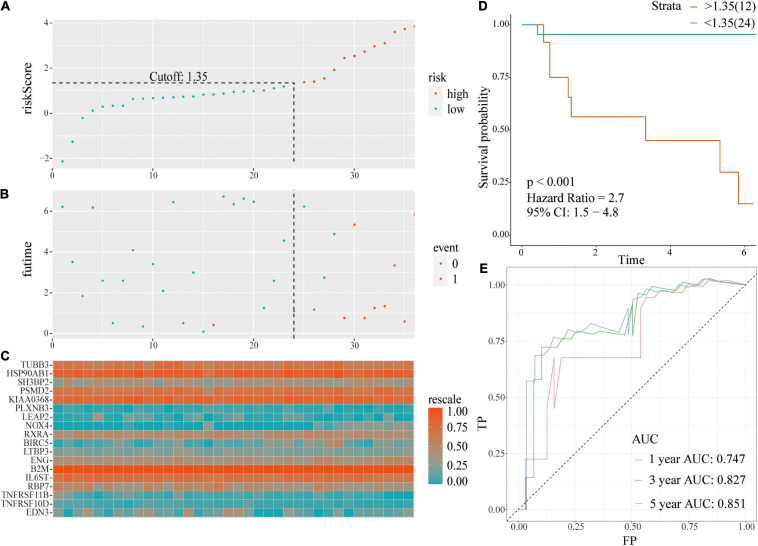
A prognostic signature based on IRGs in independent validation dataset. **(A)** Distribution of risk scores based on IRGs in PCa; **(B)** Recurrence status of patients in different groups; **(C)** Heatmap showing the expression profiles of IRGs; **(D)** Survival analysis for the signature-defined risk groups; **(E)** Time-dependent ROC curve of the 18-IRGs prognostic signature.

The same coefficient and the relative expression value of the gene were used to calculate the risk score of each patient in the external validation using GSE46602 dataset. Patients were grouped into high- or low-risk score groups based on cut-off values. The risk plot with group distribution based on signature, prognostic status of individuals between groups, and the expression level of the included IRGs are presented in Figures 5A–C. Tumor recurrence was significant higher in high-risk score groups compared with that in the low risk groups [*p* < 0.001; HR = 2.7 (1.5, 4.8)] (Figure 5D). ROC curve analysis (Figure 5E) showed good discrimination with AUC values of 0.747, 0.827, and 0.851 after 1-, 3-, and 5-year follow-up, respectively.

### Validation of Risk Score as an Independent Prognostic Factor

Univariate and multivariate Cox proportional hazards regression analysis were performed using the TCGA and GSE46602 cohorts to determine whether the risk score is an independent clinical prognostic factor (Table 3). Analysis of the TCGA cohort showed that risk score and stage are risk factors for PCa recurrence. After adjusting for stage, multivariate analysis showed that risk score was an independent prognostic factor for recurrence in PCa [HR = 2.93 (1.99–4.32), *p* < 0.001] (Table 3). Univariate analysis using GSE46602 cohort showed that risk score and distant metastasis are risk factors for PCa recurrence. After adjusting for stage, multivariate analysis showed that risk score was an independent prognostic factor for PCa recurrence [HR = 2.58 (1.31–5.12), *p* < 0.001].

**TABLE 3:** Univariate and multivariate analyses of prognostic factors in the TCGA Data set, and independent validation data set.

Characteristics		Univariate			Multivariate		
		Hazard.Ration	CI95	*P-*value	Hazard.Ration	CI95	*P*-value
TCGA dataset	Risk score	3.33	2.32–4.77	0	2.93	1.99–4.32	0
	Age	1.01	0.97–1.05	0.723	NA	NA	NA
	T_pathologic	2.9	1.68–5.02	0	1.85	1–3.42	0.048
	N_pathologic	1.97	1.08–3.59	0.026	0.99	0.52–1.88	0.978
GSE46602 dataset	Risk score	2.6	1.46–4.65	0.001	2.58	1.31–5.12	0.006
	Age	0.98	0.88–1.1	0.743	NA	NA	NA
	T_stage	13.97	1.61–121.58	0.017	9.61	1.1–83.81	0.041
	Gleason	1.32	0.68–2.57	0.417	NA	NA	NA
	PSA	0.99	0.92–1.07	0.841	NA	NA	NA

### Subgroup Prognostic Analysis Based on Multiple Classification Methods

**FIGURE 6:**
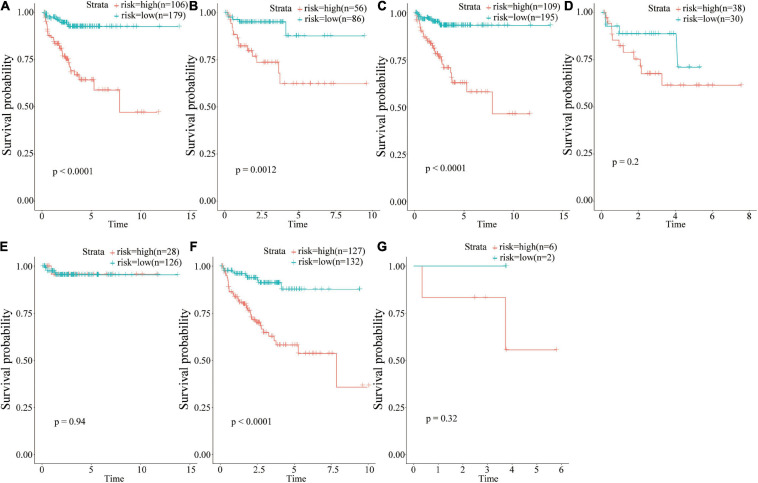
Group analysis of the prognostic value of risk scores. **(A)** <65 years age group; **(B)** ≥65 years age group; **(C)** N0 group; **(D)** N1 group; **(E)** T2 group; **(F)** T3 group; **(G)** T4 group.

The prognostic value of risk scores was determined based on 18 IRGs in different clinical groups (Figure 6). The findings showed that risk score was a potential prognostic marker in some clinical groups, including those aged <65 years (Figure 6A, *p* < 0.001), ≥65 years (Figure 6B, *p* = 0.012), N0 (Figure 6C, *p* < 0.001), and T3 (Figure 6F, *p* < 0.001). On the other hand, the risk score could not predict the prognosis in clinical groups such as N1 (Figure 6D, *p* = 0.2), T2 (Figure 6E, *p* = 0.94), and T2 (Figure 6G, *p* = 0.32).

### Gene Mutation and Transcriptome Expression Differences in the Different Risk Score Groups

**FIGURE 7:**
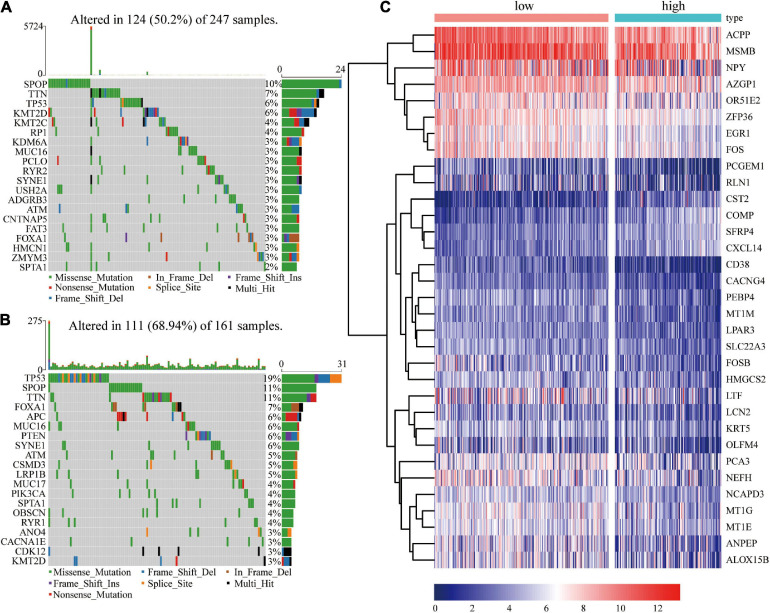
Frequency of gene mutations and differential expression of genes in different risk groups. **(A)** Visualization of gene mutations in low-risk score groups; **(B)** Visualization of gene mutations in high-risk score groups; **(C)** Heatmap showing differential gene expressions in different groups.

The findings of this study showed that risk score is an independent prognostic factor for PCa. Analysis of differences in gene mutations and transcriptome expressions between high- and low- risk groups showed that the proportions of patients with gene mutations were 50.2% (124 in 247) and 68.94% (111 in 161) in the low-risk score and high-risk score groups, respectively, (Figures 7A,B). Frequency of gene mutations in the high-risk score group was higher compared with that in the low-risk score group. Mutation frequency of TP53 (19%) was higher in the high-risk score group compared with that in the low-risk score group (6%). The frequency of SPOP mutations in the low-risk score group was 10% whereas that in the high-risk group was 11%. TTN was one of the top three genes with the highest mutation frequency in the two groups. The mutation frequency of TTN in the high-risk group (11%) was significantly higher compared with that in the low-risk group (7%).

In addition, 33 differentially expressed genes were identified between the high- and low- risk score groups (Figure 7C). Four genes (COMP, CST2, CXCL14, and SFRP4) were significant upregulated in the high-risk group, while ALOX15B, CD38, LTF, HMGCS2, ZFP36, AZGP1, and OLFM4 were significant downregulated.

### Gene Set Enrichment Analysis in the Different Risk Score Groups

**FIGURE 8:**
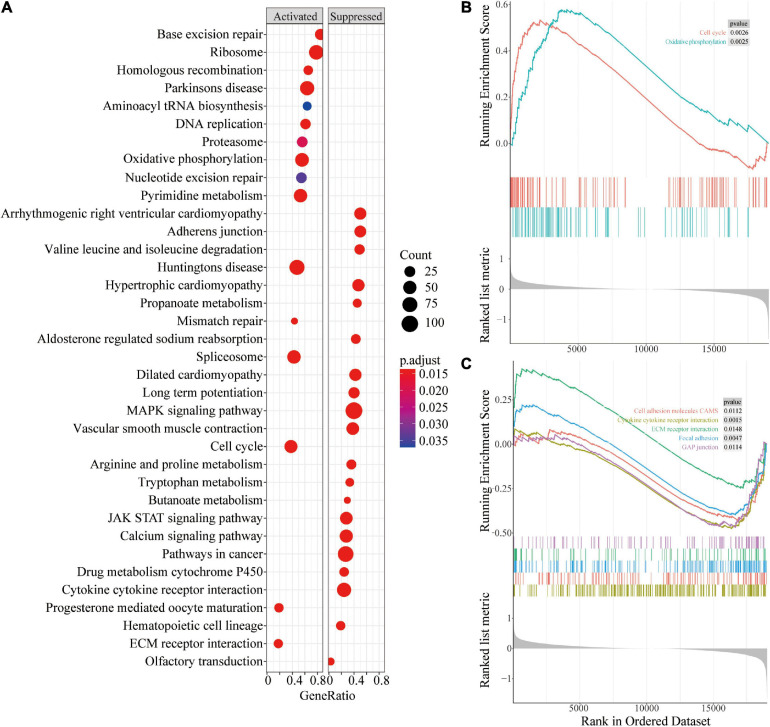
Gene Set Enrichment Analysis of different risk groups. **(A)** Bubble chart of GSEA analysis in different risk groups; **(B)** GSEA analysis of oxidative phosphorylation and cell cycle in different risk groups; **(C)** GSEA analysis of pathways related to cell connections in different risk groups.

Gene Set Enrichment Analysis (GSEA) enrichment analysis was performed to explore the potential prognostic mechanisms of risk score in PCa. The findings showed that multiple signaling pathways such as oxidative phosphorylation and cell cycle were enriched in the high-risk group (Figures 8A,B). In addition, immunity and other related signaling pathways such as cytokine-cytokine receptor interactions, cell adhesion molecules (CAMs), ECM receptor interactions, focal adhesion, and GAP junction were downregulated in the high-risk group (Figures 8A,C).

### Differences in Tumor Immune Cell Infiltration Between Different Risk Groups

**FIGURE 9:**
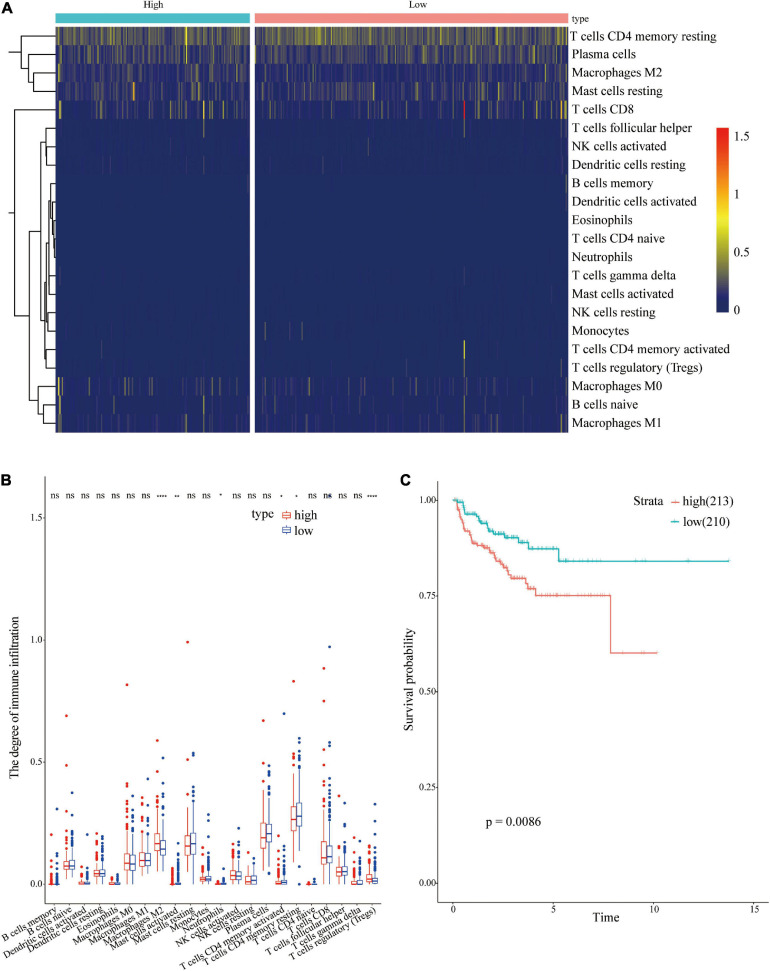
Analysis of immune cell infiltrations in different risk groups. **(A)** Heatmap showing immune cell infiltration in different risk groups; **(B)** Differential analysis of immune cells in different risk groups; **(C)** Survival analysis of Macrophages M2 in PCa.

CIBERSORTx online tool was used to evaluate immune cell infiltrations in both groups to explore the relationships between the 18 immune-related gene signatures and the immune system. The infiltration levels of Macrophage M2 and regulatory T cells (Tregs) were higher in the high-risk group compared with those in the low risk group (Figures 9A,B). However, the infiltration level of activated mast cell, Neutrophils, activated, and resting CD4 memory T cells was higher in the low risk group compared with that in the high-risk group. Univariate regression analysis and survival analysis of infiltrating immune cells showed that Macrophages M2 level was correlated with PCa prognosis (*p* = 0.0086) (Figure 9C). A high level of Macrophages M2 infiltration was correlated with poor prognosis of PCa patients. Therefore, differences in prognosis between the high and low risk groups may be attributed to differences in infiltration of Macrophages M2.

### Differences in Immuno-Regulatory Genes Between Different Risk Groups

**FIGURE 10:**
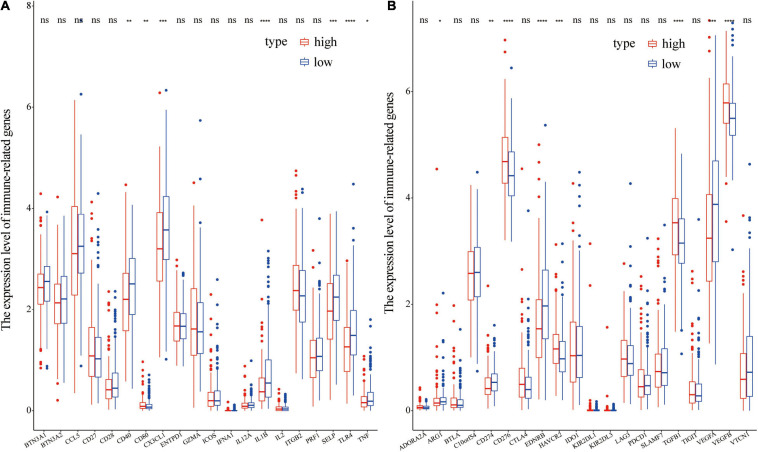
Differential expression of immunoregulatory genes in different groups. **(A)** Differential expression of immune-stimulatory genes in different groups; **(B)** Differential expression of immune-suppressive genes in different groups.

Immuno-modulatory genes such as CD40, CX3CL1, IL1B, SELP, TLR4, and TNF, circulated in low levels in the high-risk score group compared with the levels in the low-risk group (Figure 10A). However, the high-risk group showed a higher level of CD80 compared with the level in the low-risk group (Figure 10A). Levels of immune-suppressive genes such as CD276, HAVCR2, TGFB1, and VEGFB, were higher in the high-risk score group compared with the levels in the low risk group (Figure 10A). On contrary, low levels of ARG1, CD274, EDNRB, and VEGFA were observed in the high-risk group compared with the levels in the low-risk group (Figure 10B). These findings imply that differential expression of some immuno-regulatory genes may be implicated in the potential mechanism underlying differences in immune cell infiltration.

### A Nomogram for the Prediction of Prognosis in PCa Patients

**FIGURE 11:**
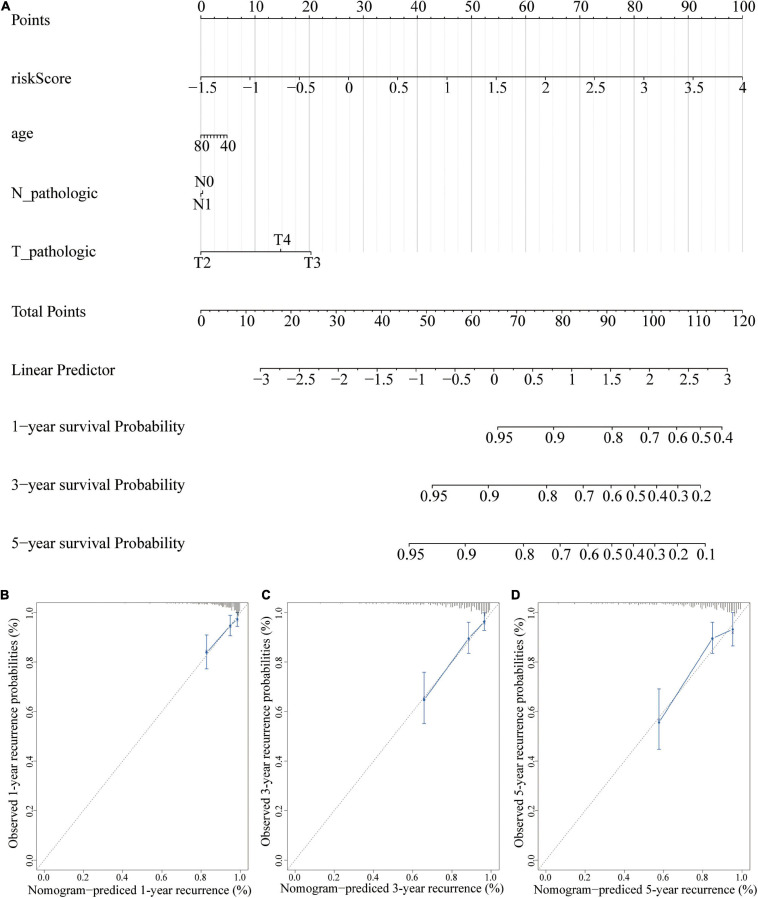
A nomogram for recurrence prediction. **(A)** Nomogram based on the signature and clinicopathological features. **(B)** Calibration plot showing that nomogram-predicted 1-year recurrence probabilities corresponded to the actual observed 1-year recurrence probabilities; **(C)** Calibration plot showing that nomogram-predicted 3-year recurrence probabilities corresponded to the actual observed 3-year recurrence probabilities; **(D)** Calibration plot showing that nomogram-predicted 5-year recurrence probabilities corresponded to the actual observed 5-year recurrence probabilities.

A nomogram is a powerful tool used to quantitatively determine individual risk in clinical environments by integrating multiple risk factors (Karakiewicz et al., 2007; Won et al., 2015). A nomogram was constructed based on risk scores, age, T, and N to predict at 1-, 3-, and 5-year prognoses. Each factor was assigned a score based on its contribution to prognosis (Figure 11A). The calibration curve showed that the actual and expected survival rates were similar (Figures 11B–D), especially the 1- and 3-year recurrence period.

### Differential Expression and Biological Role of BIRC5 Gene *in vitro*

**FIGURE 12:**
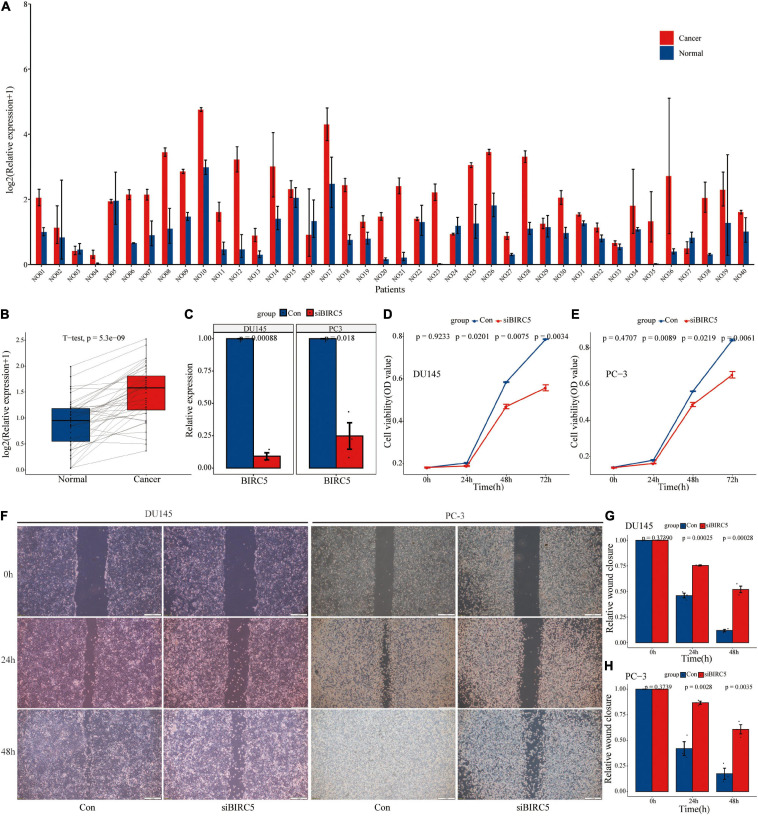
The level of BIRC5 expression in PCa tissues and its biological function. **(A,B)** The expression level of BIRC5 in 40 pairs of PCa and normal matched tissues; **(C)** RT-qPCR analysis for the transfection efficiency of BIRC5-siRNA in PC-3 and DU145 PCa cells; **(D–E)** CCK-8 assay for the proliferation of PC-3 and DU145 PCa cells after BIRC5 silencing; **(F–H)** Wound healing assays for the migration of PC-3 and DU145 PCa cells after BIRC5 silencing.

Univariate regression analysis showed that BIRC5 was a high-risk factor (HR = 1.9) of prostate cancer and its p-value was the smallest among analyzed factors (Supplementary Table 3). In addition, survival analysis showed that BIRC5 is correlated with the prognosis of prostate cancer (*p* = 0.0005852). Therefore, BIRC5 gene was chosen for further analysis of its differential expression and biological function. The findings showed that BICR5 was significantly up-regulated in 87.5% (35/40) of prostate cancer tissues compared with adjacent tissues (Figures 12A,B). BIRC5 interfering RNA was designed and synthesized, and lipo3000 was used to transfect it into prostate cells. RT-qPCR was used to determine the knockdown efficiency of interfering RNA. Interfering RNA effectively downregulated the expression level of BIRC5 (Figure 12C). After knocking down BIRC5 in prostate cancer cells, CCK-8 assay was used to determine the effect of BIRC5 on proliferation of prostate cancer cells. The findings showed that the proliferation rate of prostate cancer cells DU145 and PC-3 was significantly lower after knocking down BIRC5 compared with that of the control group (Figures 12D,E). Furthermore, the wound healing assay was used to determine the migration ability of the cells. The findings showed that the migration rate of cells was significantly reduced after knocking down BIRC5 in prostate cancer cells DU145 and PC-3 at 24 and 48h compared with that in the control group (Figures 12F–H).

In summary, the findings of this study showed that BIRC5 is highly expressed in prostate cancer tissues, and knocking down BIRC5 can significantly inhibit proliferation and migration rate of prostate cancer cells *in vitro*.

## Discussion

Prostate cancer is a common type of cancer in men and is the fifth leading cause of cancer mortalities (Bray et al., 2018). Early stages of prostate cancer are effectively treated by radical surgery or radiotherapy. However, these methods are not effective for treatment of advanced prostate cancer. The effect of resection treatment in patients with metastatic prostate cancer can only be maintained for an average of 18 months or slightly longer. Therefore, most patients transition into hormone-resistant prostate cancer. Immunotherapy stimulates the patient’s immune system to eliminate cancer cells (Farkona et al., 2016). Previous studies have explored use of immunotherapy for treatment of prostate cancer by targeting immunosuppressive molecules (De Velasco and Uemura, 2018). Sipuleucel-T (Provenge) has been approved by the FDA as an immunotherapy for treatment of metastatic castration-resistant prostate cancer. Although Sipuleucel-T improves survival outcomes of prostate cancer patients, analysis shows no significant difference in progression-free survival between the treatment and the placebo groups (Handy and Antonarakis, 2018). Efficacy of immunotherapy in prostate cancer indicates that immune-related genes play important roles in prognostic outcomes of prostate cancer patients. In addition, multigene signatures generated by reliable algorithms are accurate compared with single molecules in predicting outcomes. Therefore, there is an urgent need to explore effective immune-related biomarkers and prediction models for PCa prognosis.

Etiology of prostate cancer is complicated, therefore, a network-centric strategy, rather than a single gene/protein-centric strategy is more effective for studying cellular responses. WGCNA is highly sensitive to genes with small fold changes and is characterized by no information loss (Pei et al., 2017). In the current study, a total of 50 functional modules were identified through WGCNA analysis. Nine of these functional modules were correlated with PCa recurrence. The nine functional modules included a total of 259 IRGs. Out of the 259 IRGs, 35 IRGs were associated with PCa prognosis. An eighteen-gene signature (TUBB3, HSP90AB1, SH3BP2, PSMD2, KIAA0368, PLXNB3, LEAP2, NOX4, RXRA, BIRC5, LTBP3, ENG, B2M, IL6ST, RBP7, TNFRSF11B, TNFRSF10D, and EDN3) was constructed and effectively distinguished patients with a significantly high risk of recurrence from patients with low risk of recurrence. Univariate analysis showed that BIRC5 is a high-risk predictor in prostate cancer and its *p*-value was the smallest compared with other factors. Moreover, survival analysis showed that BIRC5 is correlated with the prognosis of prostate cancer. BIRC5, also known as Survivin plays a key role in cancer by modulating cell division and proliferation and through inhibition of apoptosis (Yamamoto et al., 2008). BIRC5 is overexpressed in most human cancers and is correlated with poor prognosis of patients, however, it is shows low expression levels in normal tissues (Duffy et al., 2007). Therapies targeting BIRC5 are considered as promising therapies for the treatment of various cancers owing to the abnormally high expression of BIRC5 during the carcinogenic process of various types of cancers. The *in vitro* experiments in the current study showed that BIRC5 is highly expressed in prostate cancer tissues, and it modulates proliferation and migration rate of prostate cancer cells. These findings show that the genes used in construction of the prognostic models play important biological roles in prostate cancer.

The findings of the current study showed that risk score was an independent prognostic factor for prostate cancer patients after adjusting other factors. In addition, risk score accurately predicted prognosis of patients in the <65-year age group, ≥65-year age group, N0 group, and T3 group. Notably, risk score showed poor predictive ability in other groups. This can be attributed to the small sample size. Nomogram analysis showed that combining this model with other clinical features can improve its 1- and 3-year prognostic accuracy. Therefore, the model constructed in this study can be used to identify prostate cancer patients with a high risk of recurrence, thus ensuring early intervention to improve outcomes.

Gene mutation analysis, transcriptome difference analysis, and immune infiltration analysis were conducted to explore the potential mechanism of the prognosis model based on 18 IRGs. The findings showed that patients in the high-risk group had higher gene mutations compared with patients in the low-risk group. TP53 gene showed the highest mutation frequency in the high-risk group, with missense mutations as the dominant mutations. The mutation frequency of TP53 gene was significantly higher compared with that of the low-risk group. Notably, TP53 mutations are higher in CRPC compared with hormone-sensitive cancer stage. Thus, TP53 mutations promote the development of castration resistance in prostate cancer (Robinson et al., 2015; Lei et al., 2018). High-frequency mutations in TP53 may explain the poor prognosis in high-risk groups.

In addition, transcriptome differential analysis was performed for high and low risk groups and 33 differentially expressed genes were identified. ALOX15B, CD38, LTF, HMGCS2, ZFP36, AZGP1, and OLFM4 showed low expression levels in the high-risk group compared with the levels in the low-risk group. ALOX15B is a human-specific lipid peroxidase and is significantly highly expressed in the epithelial cells of the normal human prostate. However, ALOX15B is downregulated or completely absent in more than 70% of PCa cases, exerting a tumor suppressive function (Suraneni et al., 2014). Expression of CD38 was inversely correlated with PCa progression. Low CD38 is implicated in decrease of intracellular NAD + levels in PCa cells, leading to cell cycle arrest. Notably, CD38 suppreses glycolysis and mitochondrial metabolism, activates AMP-activated protein kinase (AMPK), and inhibits synthesis of fatty acids and lipids. Loss of CD38 confers a metabolic advantage during carcinogenic transformation of prostate cancer (Chmielewski et al., 2018). Differentially expressed genes in high and low risk groups affect prognosis of prostate cancer patients. GSEA analysis of all genes in the high- and low-risk groups showed that pathways associated with intercellular junctions, such as cell adhesion molecules CAMs, ECM receptor interaction, focal adhesion, and GAP junction were enriched in high-risk groups.

Immune characterization of prostate cancer patients showed that level of infiltration of Macrophages M2 and T cells regulatory (Tregs) was higher in the high-risk group compared with that in the low-risk group. Level of activated mast cells, Neutrophils, CD4 memory activated T cells, and CD4 memory resting T cells were lower in the high-risk group compared with the levels in the low risk group. Tumor-associated macrophages are divided into M1 phenotype which inhibits tumors or M2 phenotype which promotes tumor formation. M2 macrophages are associated with poor clinical outcomes as they stimulate angiogenesis, metastasis, and immunosuppression (Solinas et al., 2009; Shalapour and Karin, 2015; Erlandsson et al., 2019). Regulatory T cells promote tumor development by inhibiting anti-tumor immunity (Chen et al., 2016). Previous studies report that high number of Tregs in PCa patients is associated with poor prognosis and can reduce survival of Pca patients (Ebelt et al., 2009; Valdman et al., 2010; Davidsson et al., 2018). Survival analysis showed that M2 macrophages were associated with tumor recurrence in patients with prostate cancer. Therefore, M2 macrophages may be a potential factor affecting prognosis of prostate cancer patients in the high and low risk groups. Moreover, the findings of this study showed that immuno-modulatory genes, such as CD40, CX3CL1, IL1B, SELP, TLR4, and TNF, were downregulated in the high-risk group, whereas immune-suppressive genes, such as CD276, HAVCR2, TGFB1, and VEGFB, were upregulated in the high-risk group. Differential expression of immune regulatory genes may be correlated with prognosis of prostate cancer patients in the high and low risk groups. However, further studies should be conducted to fully explore the mechanism underlying the effect of immune related genes on prognosis of PCa patients.

The findings from the current study were consistent with findings from previous studies (Rui et al., 2019; Liu et al., 2020; Luan et al., 2021). The one with a higher degree of similarity was the paper by Liu et al. (2020). However, the current study had a few advantages compared with the study by Liu et al. (2020). First, in the current study, prostate cancer patients in TCGA datasets were randomly divided into two cohorts (training cohort and the testing cohort) at a ratio of 7:3. The training cohort was used to build the prognostic model, and the testing cohort was used to test the prognostic model. In addition, external validation was performed using the GEO database. GSE46602 dataset was used as an external dataset for external verification of the prognostic model. However, in the study by Liu et al. (2020), a training cohort (GSE54460) and the TCGA cohort were only used as datasets. This implies that the reliability of the findings of the current study is higher. Furthermore, the AUC value of the prognostic model of the current study is higher compared with that reported by Liu et al. (2020) ROC curve analysis using the TCGA-training set in the current study showed a good discrimination of the model with AUCs of 0.768, 0.769, and 0.741 after 1-, 3-, and 5-year follow-up, respectively. ROC curve analysis using the TCGA-testing set in the current study showed acceptable discrimination with AUCs of 0.753, 0.814, and 0.829 after 1-, 3-, and 5-year follow-up, respectively. Further, ROC curve analysis using the external validation GSE46602 dataset in in the current study showed acceptable discrimination with AUCs of 0.747, 0.827, and 0.851 after 1-, 3-, and 5-year follow-up, respectively. However, in the study by Liu et al. (2020) the AUC values were 0.749 at 1 year, 0.804 at 3 years, and 0.774 at 5 years for the GSE54460 cohort whereas the AUC values were 0.644 at 1 year, 0.69 at 3 years, and 0.691 at 5 years for the TCGA cohort. These findings show that the model in the current study was more accurate compared with that reported previously (Liu et al., 2020). In the current study, a nomogram was generated to predict prognoses of year 1, 3, and 5 by combining risk scores, age, T, and N. However, the study by Liu et al. (2020) did not construct a nomogram. In summary, the findings of the current study are more reliable compared with findings from previous studies.

## Conclusion

In the current study, a risk model was constructed based on 18 IRGs for accurate prediction of prognosis of prostate cancer patients. The findings showed that the risk scores generated by the established model were an independent prognostic indicator of prognosis of PCa patients. In addition, patients with different risk groups exhibited significant differences in gene mutations, differentially expressed genes and immune infiltration, which may be potential mechanisms underlying the differences in prognosis of patients in these groups. However, further studies should be conducted to validate the findings of the current study.

## Data Availability Statement

The original contributions presented in the study are included in the article/Supplementary Material, further inquiries can be directed to the corresponding authors.

## Ethics Statement

The studies involving human participants were reviewed and approved by The Ethics Committee of Shandong Provincial Hospital. The patients/participants provided their written informed consent to participate in this study.

## Author Contributions

MF performed the experiments and revised the manuscript. ZC and XJ designed the study. WK and QW wrote the manuscript, collected the data, performed data analysis, interpretation, and revised the final manuscript. HW and YD collected the data and performed data analysis. JW performed data analysis. All authors read and approved the final manuscript.

## Conflict of Interest

The authors declare that they have no known competing financial interests or personal relationships that could have appeared to influence the work reported in this paper.

## Publisher’s Note

All claims expressed in this article are solely those of the authors and do not necessarily represent those of their affiliated organizations, or those of the publisher, the editors and the reviewers. Any product that may be evaluated in this article, or claim that may be made by its manufacturer, is not guaranteed or endorsed by the publisher.

## Footnotes

1
https://www.ncbi.nlm.nih.gov/geo/

2
https://immport.niaid.nih.gov

3
https://www.gsea-msigdb.org/gsea/downloads.jsp

4
https://cibersortx.stanford.edu/index.php

5
https://www.R-project.org
